# The normalizing effects of the CYP46A1 activator efavirenz on retinal sterol levels and risk factors for glaucoma in *Apoj*^*−/−*^ mice

**DOI:** 10.1007/s00018-023-04848-y

**Published:** 2023-07-01

**Authors:** Nicole El-Darzi, Natalia Mast, Yong Li, Brian Dailey, Min Kang, Douglas J. Rhee, Irina A. Pikuleva

**Affiliations:** Department of Ophthalmology and Visual Science, Case Western Reserve University, Cleveland, OH 44106 USA

**Keywords:** APOJ, Retina, Cholesterol, Efavirenz, Glaucoma, 24-hydroxycholesterol

## Abstract

Apolipoprotein J (APOJ) is a multifunctional protein with genetic evidence suggesting an association between *APOJ* polymorphisms and Alzheimer’s disease as well as exfoliation glaucoma. Herein we conducted ocular characterizations of *Apoj*^*−/−*^ mice and found that their retinal cholesterol levels were decreased and that this genotype had several risk factors for glaucoma: increased intraocular pressure and cup-to-disk ratio and impaired retinal ganglion cell (RGC) function. The latter was not due to RGC degeneration or activation of retinal Muller cells and microglia/macrophages. There was also a decrease in retinal levels of 24-hydroxycholesterol, a suggested neuroprotectant under glaucomatous conditions and a positive allosteric modulator of N-methyl-d-aspartate receptors mediating the light-evoked response of the RGC. Therefore, *Apoj*^*−/−*^ mice were treated with low-dose efavirenz, an allosteric activator of CYP46A1 which converts cholesterol into 24-hydroxycholesterol. Efavirenz treatment increased retinal cholesterol and 24-hydroxycholesterol levels, normalized intraocular pressure and cup-to-disk ratio, and rescued in part RGC function. Retinal expression of *Abcg1* (a cholesterol efflux transporter), *Apoa1* (a constituent of lipoprotein particles), and *Scarb1* (a lipoprotein particle receptor) was increased in EVF-treated *Apoj*^*−/−*^ mice, indicating increased retinal cholesterol transport on lipoprotein particles. Ocular characterizations of *Cyp46a1*^*−/−*^ mice supported the beneficial efavirenz treatment effects via CYP46A1 activation. The data obtained demonstrate an important APOJ role in retinal cholesterol homeostasis and link this apolipoprotein to the glaucoma risk factors and retinal 24-hydroxycholesterol production by CYP46A1. As the CYP46A1 activator efavirenz is an FDA-approved anti-HIV drug, our studies suggest a new therapeutic approach for treatment of glaucomatous conditions.

## Introduction

Apolipoprotein J (APOJ) or clusterin is an evolutionary conserved multi-functional protein with a mouse to human identity of 81% and similar tissue distribution in the two species [1–3]. Normally, APOJ is a secreted protein but cytoplasmic and nuclear APOJ forms also exist and can be present under certain conditions [4, 5]. Functionally, APOJ has the capacity to bind different types of molecules including denatured proteins, cholesterol, phospholipids, complement components, immunoglobulins, amyloid β fibrils, and different receptors [4, 6]. Accordingly, APOJ is a chaperone, which can participate in lipid transport, complement regulation, apoptosis, barrier function, cytoprotection, and neurodegeneration [3, 6–11]. Studies showed that APOJ limits inflammatory injury and that the transcriptional regulator heat shock factor 1 increases the *APOJ*/*Apoj* expression. These findings led to a notion that APOJ is a protective molecule upregulated during times of physiological stress [10, 12–14].

*APOJ* is the third largest genetic risk factor for late onset Alzheimer’s disease (e.g., rs11136000, rs2279590, and rs9331888) after *APOE* and *BIN1*, accounting for 27% of late onset disease heritability [15, 16]. This is due to the SNPs that decrease protein expression; conversely, the SNPs that increase APOJ expression are protective against the disease [17, 18]. In addition, several APOJ SNPs (rs3087554 and rs2279590) have been nominally associated with exfoliation syndrome (XFS) and the associated glaucoma (XFG) in certain ethnic groups [19].

In the eye, APOJ is expressed in various parts including trabecular meshwork and retina, the former being a sensory organ in the back of the eye [6, 20–22]. In both ocular tissues, APOJ was suggested to play a protective role as indicated by cell culture studies and animal models of retinal ischemia, diabetic retinopathy, light-induced retinal degeneration, and retinitis pigmentosa [22–30]. Yet, the APOJ protective effects are not firmly established, and the underlying mechanisms of these effects are still not clear.

The retina is rich in cholesterol and maintains cholesterol homeostasis by balancing the pathways of cholesterol input and output [31, 32]. Retinal cholesterol input includes local biosynthesis and uptake of cholesterol-containing lipoprotein particles (LPP) from the systemic circulation. Retinal cholesterol output is realized via photoreceptor phagocytosis, metabolism to oxysterols (27-hydroxycholesterol, 27HC; 5-cholestenoic acid, 27COOH; 7α-hydroxy-3-oxo-4-cholestenoic acid, 7HCA; and 24-hydroxycholesterol, 24HC) by cytochrome P450 enzymes 27A1 and 46A1 (CYP27A1 and CYP46A1, respectively) as well as transport on LPP to the systemic circulation [31, 32]. In addition, a small fraction of retinal cholesterol (up to 15%) is esterified [33, 34] either for storage or as a part of transport on LPP. Three enzymes, SOAT1, SOAT2 (sterol-O-acyltransferases 1 and 2, respectively), and LCAT (lecithin-cholesterol acyltransferase) can potentially esterify cholesterol in the retina [35–40]. Elaborate mechanisms coordinate retinal cholesterol input, transport, storage, and output to maintain the steady-state sterol levels [31, 32, 38, 41].

Previously we established that APOJ along with apolipoproteins E, A1, and A4 (APOE, APOA1, and APOA4, respectively) are abundant in mouse retina [42], where they are the constituents of retinal LPP, proposed to circulate in the intraretinal space and transport cholesterol between different retinal cells [43, 44]. We also found that retinal expression of APOAs, APOE and a less abundant APOD seems to be interdependent, whereas that of APOJ appears to be unaffected by the expression of other apolipoproteins [42, 45]. Like in the brain, this could be due to integration of APOJ into retinal LPP different from those that contain APOA1 and APOE [46, 47], thus enabling LPP that contain different apolipoproteins to have overlapping but not identical functions and serve different types of retinal cells.

(*S*)-Efavirenz (EFV) is an FDA-approved anti-HIV drug that we discovered interacts off target with the CNS-specific enzyme CYP46A1 and allosterically activates this enzyme at a low dose (0.1 mg/kg body weight) in the brain and retina of mice. CYP46A1 activation by EFV was found to increase tissue levels of the biologically active sterol 24HC, the rate of tissue cholesterol turnover [48–50], and mitigate the disease manifestations in mouse models of Alzheimer’s disease, depression, Niemann-Pick disease type C, glioblastoma, and prion disease [48, 49, 51–54]. In addition, in a model of Alzheimer’s disease (5XFAD mice), EFV treatment had an ocular effect, namely reduced the frequency and leakage from retinal vascular lesions [50]. A model of how activation of one enzyme can benefit different diseases and affect multiple processes was proposed [55, 56]. Importantly, low dose EFV was already confirmed to be safe and activate CYP46A1 in the brain of human subjects with early Alzheimer’s disease [57]. Herein, we conducted comprehensive ocular characterization of *Apoj*^*−/−*^ mice and obtained novel important insights into the role of APOJ as a retinal cholesterol carrier and protein required for retinal function. Also, we documented the beneficial EFV effects on glaucoma risk factors in *Apoj*^*−/−*^ mice. This pre-clinical study justifies further ocular investigations of EFV in human subjects with glaucoma risk factors and/or Alzheimer’s disease.

## Materials and methods

### Animals and EFV treatment

*Apoj*^*−/−*^ mice on the C57BL/6J background [12, 58] were obtained from Dr. P. Pattabiraman (Indiana University, Indianapolis, Indiana, USA). *Cyp46a1*^*−/−*^ mice on the mixed C57BL/6J;129S6/SvEv background [59] were provided by Dr. David Russell (UT Southwestern, Dallas, TX) and backed crossed for 10 generations to the C57BL/6 J background. All animals were free of the *Crbl*^*rd8*^ mutation. Mice were maintained on a standard 12-h light (∼10 lx)-dark cycle with food and water ad libitum. For EFV treatment, littermates were selected from the pool of all available animals and randomly assigned to either the control or treatment group, which were matched by size and sex. No statistical methods were used to predetermine sample size. EFV (Toronto Research Chemicals, North York, ON M3J 2K8, Canada, E425000) was administered in drinking water containing 0.0004% Tween 80 at a 0.1 mg/kg body weight/day dose from 3 to 7 months of age. Control group received drinking water containing 0.0004% Tween 80. The investigators were not blinded with respect to EFV treatment as they were involved in both animal treatment and subsequent studies of retinal sterols, intraocular pressure (IOP), and pattern electroretinography (PERG) recordings. All animal experiments were approved by Case Western Reserve University IACUC and conformed to the recommendations of the American Veterinary Association Panel on Euthanasia.

### In vivo imaging

Retinal imaging by fundus photography, ultra-high resolution spectral domain optical coherence tomography (SD-OCT), and fluorescein angiography (FA) was carried out as described [60–62]. An Ivivo fundoscope (Xenotec OcuScience, Henderson, NV, USA), Envisu R2200 UHR OCT imaging system (Leica Bioptigen, Morrisville, NC, USA), and a scanning laser ophthalmoscope (Spectralis HRA, Heidelberg Engineering, Franklin, MA, USA) were used, respectively, for each imaging modality. The total thickness of the retina and retinal layers was measured automatically by the Diver software of the OCT imaging system. Images for FA were acquired after a bolus (0.1 ml) intraperitoneal injection of 1.0% sodium fluorescein (Akorn Inc., Lake Forest, IL, USA, #17478-250-20) in phosphate buffer saline (PBS).

### IOP measurements

These measurements were carried out as described [22] after mice were anesthetized via intraperitoneal injection of 80 mg/kg ketamine and 15 mg/kg xylazine (Patterson Veterinary, Devens, MA, USA, 07-890-85598 and 07-808-1947, respectively) in sterile distilled water. From 4 to 7 min post injection, a validated commercial rebound tonometer (TonoLab; Colonial Medical Supply, Londonderry, NH, USA) was used to take three sets of six measurements of IOP in each eye. The tonometer was fixed horizontally for all measurements with the tip of the probe being 2–3 mm from the eye. The probe contacted the eye perpendicularly over the central cornea, and a set of measurements was accepted only if the device indicated that there was no significant variability. All the measurements were taken between 11 AM and 3 PM [22].

### PERG recordings

Mice were dark-adapted overnight, and the next morning anesthetized via intraperitoneal injection of 80 mg/kg ketamine and 15 mg/kg xylazine. Animals were placed under a dim red light on a heated pad (37◦C), and tropicamide (1% eye drops; Akorn Inc. Lake Forest, IL, USA) was applied to reduce eye sensitivity and dilate the pupils. The Celeris Model D430 system (Diagnosys LLC, Lowell, MA, USA) was used. The pattern stimulator was placed on one eye, and the flash stimulator was placed on the contralateral eye to serve as the reference electrode. Transient PERG responses were recorded using black and white vertical stimuli at 50 cd·s/m^2^ luminance, and then 400 hundred PERG waveforms were averaged with the cut-off filter frequencies from 1 to 50 Hz.

### Retinal sterol measurements

Individual or pooled samples of mouse retina containing retinal pigment epithelium (RPE) were used. These samples were supplemented with deuterated sterol analogs, which served as internal standards, for sterol quantifications by isotope dilution gas chromatography-mass spectroscopy. [34, 61]. The amount of total cholesterol represented a sum of esterified and unesterified cholesterol (EC and UC, respectively).

### Retinal histochemistry

Lipid distribution in the retina was assessed as described [41, 63, 64] by stains with filipin (Cayman Chemical, Ann Arbor, MI, USA, #70440) for UC and Bodipy 493/503 (ThermoFisher Scientific, Inc., Waltham, MA, USA, D3922) for UC, EC, triacylglycerides, and free fatty acid.

### Retinal immunohistochemistry

Frozen retinal sections were prepared as described [61]. Double stains for BRN3A and NeuN were conducted as described for BRN3A [62]. The following sets of primary and secondary antibodies were used: the primary rabbit monoclonal antibody to BRN3A (Abcam, Waltham, MA, USA, ab245230, 1:200 dilution) and goat anti-rabbit Alexa Fluor 647 secondary antibody (Jackson ImmunoResearch, West Grove, PA, USA, 111-605-144, 1:200 dilution); chicken polyclonal primary antibody to NeuN (Synaptic Systems, Goettingen, Germany, #266006, 1:500 dilution) and goat anti-chicken Alexa Fluor 488 secondary antibody (ThermoFisher Scientific, A11039, 1:200 dilution). Double stains for glial fibrillary acidic protein (GFAP) and ionized calcium binding adaptor molecule (Iba1) were as described [42] and used chicken anti-GFAP primary antibody (Thermo Fisher Scientific, PA1-10004, 1:500 dilution) and donkey anti-chicken Alexa Fluor 594 secondary antibody (Jackson Immune Res Inc. #703-585-155, 1:200); rabbit monoclonal antibody to Iba1 (Abcam ab178846, 1:250 dilution) and goat anti-rabbit Alexa Fluor 647 secondary antibody (Jackson ImmunoResearch, West Grove, PA, USA, 111-605-144, 1:200 dilution). After incubation with secondary antibodies, slides were washed two times with PBS, then with distilled water, and incubated at room temperature for 10 min in a solution of 0.035% (W/V) Sudan black (ThermoFisher Scientific, #4197-25-5) in 70% ethanol as described [41] to reduce autofluorescence [65]. Double stains for APOJ and glutamine synthetase were as described for glutamine synthetase [60], except the blocking buffer contained 10% normal goat serum (Life Technologies, PCN5000), and these stains included treatment with 0.1% (W/V) Sudan black. Rabbit monoclonal primary antibody to clusterin-α chain (Abcam ab230150, 1:250 dilution) and goat anti-rabbit Alexa Fluor 647 (Jackson ImmunoReasearch, 111-605-144, 1:200 dilution) as well as guinea pig polyclonal primary antibody to glutamine synthetase (Synaptic Systems, #367005, 1:500 dilution) and goat anti-guinea pig Alexa Fluor 568 secondary antibody (ThermoFisher Scientific, A11075, 1:200 dilution) were used. All slides were imaged on a Zeiss AxioScan.Z1 (Carl Zeiss Research Microscopy Solutions, White Plains, NY, USA) equipped with the high performance Hamamatsu ORCA Flash4.0 v3 monochrome camera and the 20x/0.8 Plan-Apochromat objective, and a Colibri 7 Illumination kit. In addition, stains for APOJ and glutamine synthetase were imaged on an Olympus Fluoview FV1200 Laser Scanning Confocal Microscope (Olympus, Center Valley, PA 18034, USA).

### Data and statistical analysis

All images are representative of studies in three to five animals per genotype unless otherwise indicated. To minimize investigators’ bias in non-quantitative assessments, retinal regions that were compared were matched by the retinal location and animal age. All quantitative data represent the mean ± SD and were analyzed either by two-way ANOVA with Tukey’s multiple comparison test, unpaired non-parametric Mann–Whitney test or a two-tailed unpaired Student’s t test. The sample size (n) is indicated in each figure or figure legend. Statistical significance was defined as *, *P* ≤ 0.05; **, *P* ≤ 0.01; ***, *P* ≤ 0.001. The quantitative studies were not affected by investigators’ bias as all data were used and apparent outliers were not excluded.

### Retinal proteomics

The label-free approach was used as described [42] and was carried out by the Proteomics and Small Molecule Mass Spectrometry Core at Case Western Reserve University (Cleveland, OH, USA). Four biological replicates per genotype, each representing a pooled sample of three retinas from three different 6-month old male mice were used. Differences in relative protein abundance were calculated by the PEAKS software (Bioinformatics Solutions Inc. Waterloo, ON, Canada) based on unique peptides [66]. Proteins with non-significant changes (*P* > 0.05) in abundance between the groups were excluded from the subsequent analysis as were the proteins with less than 1.2-fold change in the relative abundance, even if this change was significant.

## Results

### Retinal APOJ immunolocalization

**Fig. 1:**
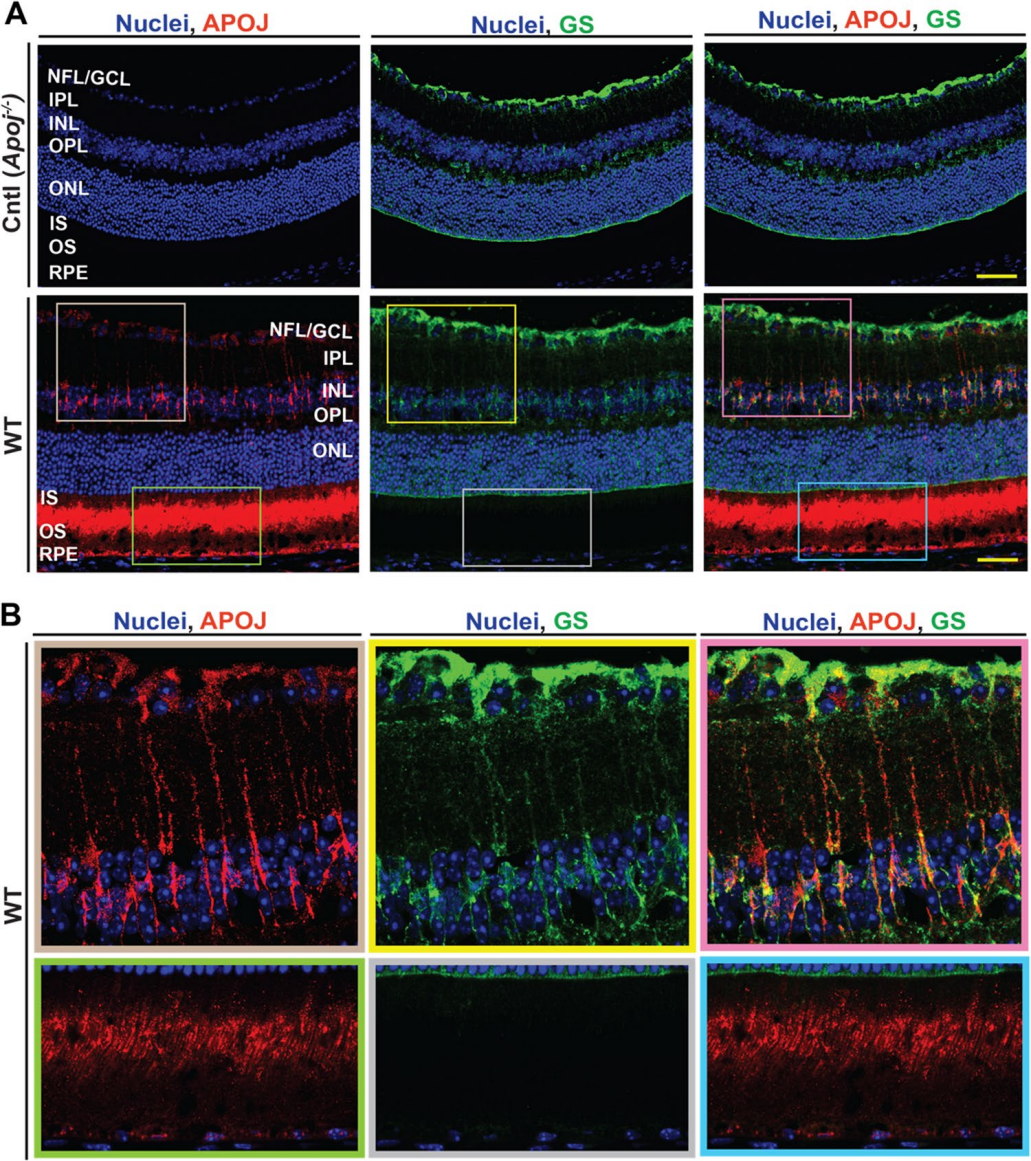
APOJ localization in the wild type (WT) retina. **A** Representative stains (3 female and 3 male per genotype) for APOJ (red) and glutamine synthetase (GS, green), a marker for non-activated Muller cells. *Apoj*^*−/−*^ mice were used as a negative control (Cntl). Nuclei were stained with DAPI (blue). Yellow scale bars are 50 μm. **B** Enlarged images of the boxed regions in **A**. The fluorescence in the upper panels was artificially enhanced as compared to that in the corresponding boxed regions in panel **A**, and fluorescence in the lower panels was decreased as compared to panel **A**. *NFL* nerve fiber layer; *GCL* the ganglion cell layer; *IPL* the inner plexiform layer; *INL* the inner nuclear layer; *OPL* the outer plexiform layer; *ONL* the outer nuclear layer; *IS* the photoreceptor inner segments; *OS* the photoreceptor outer segments; *RPE* retinal pigment epithelium

Previous immunolocalizations of APOJ in the retina by others produced inconsistent results [25–27, 29, 30, 67]. Therefore, to better interpret the data, we conducted APOJ immunolocalization in the present work and used *Apoj*^*−/−*^ retina as a negative control. In WT mice, the strongest APOJ immunoreactivity was observed in the photoreceptors (probably in the regions of inner segments (IS) and outer segments (OS) adjacent to the cilium) as well as the RPE (Fig. 1A). In addition, a weaker signal was present around some of the nuclei in the GCL as well as the cells whose shape was indicative of Muller cells, which span across the retina (Fig. 1B). Hence, slides were additionally stained for glutamine synthetase, expressed constitutively in Muller cells [68]. Merging of the two stains reveals signal co-localization in the Muller cell somas (inner nuclear layer, INL) and Muller cell end feet (nuclear fiber layer, NFL, Fig. 1B). This staining pattern confirmed some of the earlier studies demonstrating the APOJ localizations in the NFL/ganglion cell layer, GCL [26, 67], Muller cells [20, 27, 30], and photoreceptors [30].

### Retina sterol quantifications

**Fig. 2:**
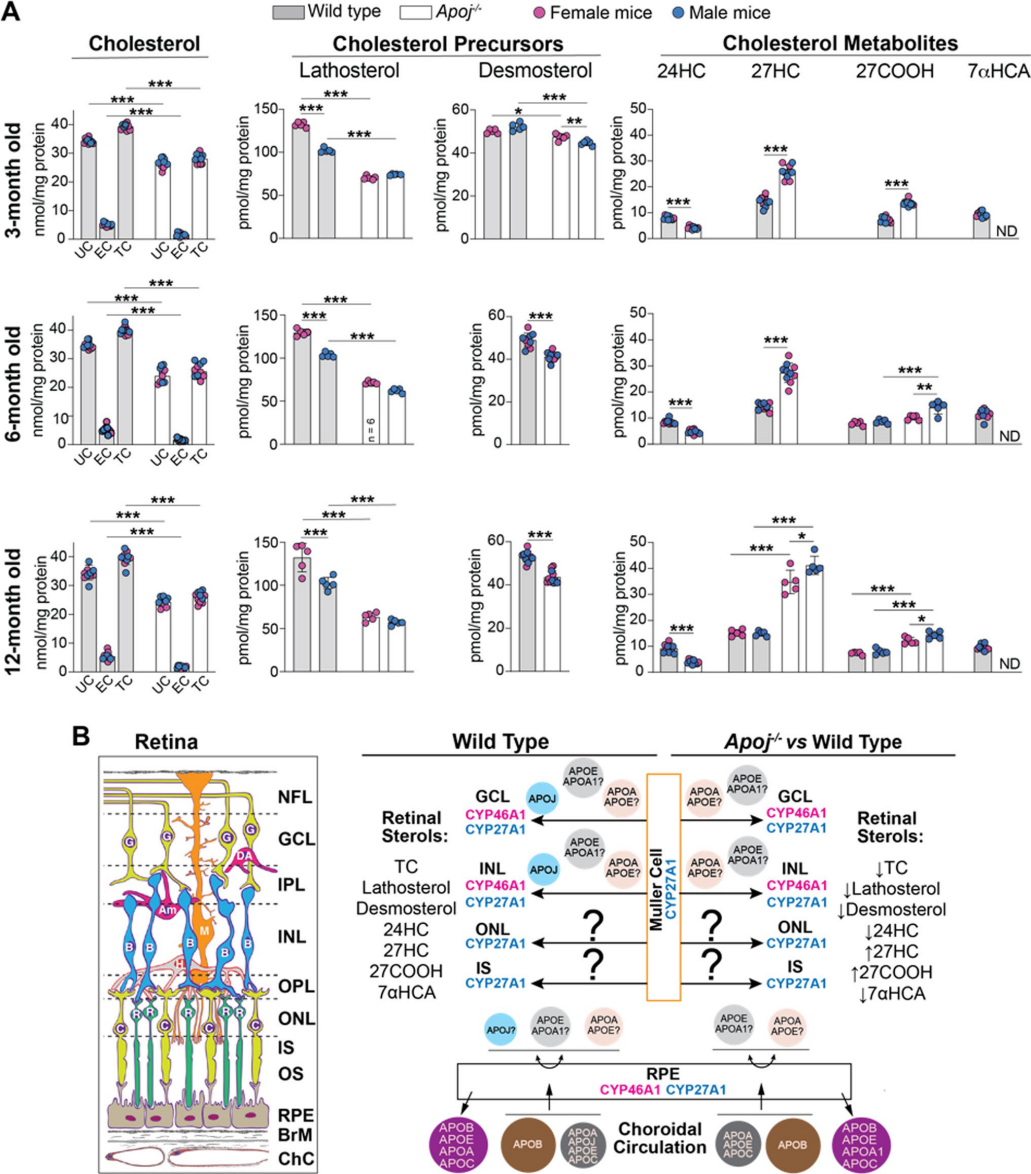
Retinal sterols in wild type and *Apoj*^*−/−*^ mice. **A** Sterol content in mice of different ages. Data represent the mean ± SD of the measurements either in individual retinas (cholesterol, lathosterol, and desmosterol: 5–6 female and 5–6 male mice per each sterol group) or in pooled samples, each containing 2 retinas from 2 different animals of the same age and sex (all cholesterol metabolites: 5 samples from female and 5 samples from male mice per each metabolite). *P ≤ 0.05; **P ≤ 0.01; ***P ≤ 0.001 as assessed by two-way ANOVA with Tukey's multiple comparison test (for all sterols). In cases where no statistical significance was found between female (magenta circles) and male mice (blue circles), the data for both sexes were combined; otherwise they were presented separately. **B** Schematic summary of sterol measurements in different experimental groups. Retinal structure (inset), CYP localizations to retinal layers, and proposed apolipoprotein particles that carry cholesterol from Muller cells to different retinal layers as well as circulate between the choroid and RPE (retinal pigment epithelium) are also shown. The question marks near apolipoproteins A1 and E indicate that it is not known whether the two proteins are present on the same or different lipoprotein particles. The retinal layer labeling is as described in Fig. 1. 24-Hydroxycholesterol, 24HC; 27-hydroxycholesterol, 27HC; 5-cholestenoic acid, 27COOH; 7α-hydroxy-3-oxo-4-cholestenoic acid, 7HCA; *Am* amacrine cells; *B* bipolar cells; *BrM* Bruch’s membrane; *C* cones; *ChC* choroidal circulation; *DA* displaced amacrine cells; *EC* esterified cholesterol; *G* ganglion cells; *M* Muller cell; *ND* not detectable (the limit of detection is 1 pmol/mg protein); *R* rods; *TC* total cholesterol; and *UC* unesterified cholesterol. The question marks indicate that it is not yet known whether there is cholesterol transport from retinal Muller cells to the photoreceptor somas and inner segments. Upwards (↑), downwards (↓), and left–right ( ↔) arrows indicate increases, decreases, and no change in sterol content, respectively, relative to the indicated comparison group. Retinal structure was taken from [41]

To assess whether APOJ contributes to retinal cholesterol homeostasis, retinal sterols were measured in 3-, 6-, and 12-month old wild type (WT) and *Apoj*^*−/−*^ mice. In both genotypes, the levels of UC, EC, and total cholesterol remained the same at all tested ages and were lower in *Apoj*^*−/−*^* vs* WT mice for all sterol forms (UC: 24–27 *vs* 34–35 nmol/mg protein; EC: 2 vs 5 nmol/mg protein; and total cholesterol: 26–28 *vs* 39–40 nmol/mg protein) (Fig. 2A). Thus, we documented that APOJ is an important player in retinal cholesterol maintenance.

To gain mechanistic insights, retinal content of cholesterol precursors lathosterol (a marker of cholesterol biosynthesis in neurons) and desmosterol (a marker of cholesterol biosynthesis in astrocytes) was quantified [69, 70]. The levels of both precursors were decreased in *Apoj*^*−/−*^* vs* WT mice at all ages, and decreases in the lathosterol levels (females: 63–71 *vs* 130–132 pmol/mg protein; and males 57–74 *vs* 102–103 pmol/mg protein) were higher than those in the desmosterol levels (females: 42–48 *vs* 49–53 pmol/mg protein; and males 41–45 *vs* 49–53 pmol/mg protein) (Fig. 2A). Apparently, lack of APOJ mostly decreased cholesterol biosynthesis in retinal neurons than in astrocytes. Next, we measured the content of retinal cholesterol metabolites. These were 24HC, mostly produced in neurons as CYP46A1 is normally a neuronal enzyme, and 27HC along with 27COOH and 7HCA, generated by the ubiquitous CYP27A1, which was previously mostly immunolocalized to retinal Muller cells, NFL/GCL, INL, and IS (Fig. 2B) [41, 71–73]. The overall production of 24HC was reduced from 8–9 pmol/mg protein in WT mice to 4–5 pmol/mg in *Apoj*^*−/−*^ mice of 3–12 months of age, whereas that of 27HC and 27COOH was increased from 14–15 to 26–38 pmol/mg protein and from 7–8 to 12–14 pmol/mg protein, respectively (Fig. 2A,B). Retinal levels of 7HCA, the downstream product of CYP27A1, became undetectable. These results were consistent with the notion [20] that APOJ likely carries cholesterol from retinal Muller cells, which express CYP27A1 (Fig. 2B) [41, 73], to retinal neurons, some of which express CYP46A1 [41, 71, 72]. If so, lack of cholesterol transport on APOJ-containing LPP should increase cholesterol levels and hence metabolism by CYP27A1 in Muller cells and consequently decrease cholesterol levels and metabolism by CYP46A1 in retinal neurons (Fig. 2B). In the latter, cholesterol biosynthesis would be decreased as well as it is coupled to cholesterol 24-hydroxylation [59]. The levels of EC (a storage form of excess cholesterol) should also decrease to compensate in part for the insufficient cholesterol supply. We observed all these predicted effects on retinal sterols in agreement with the predicted APOJ function as a retinal cholesterol career.

### Retinal cholesterol and lipid distribution

**Fig. 3:**
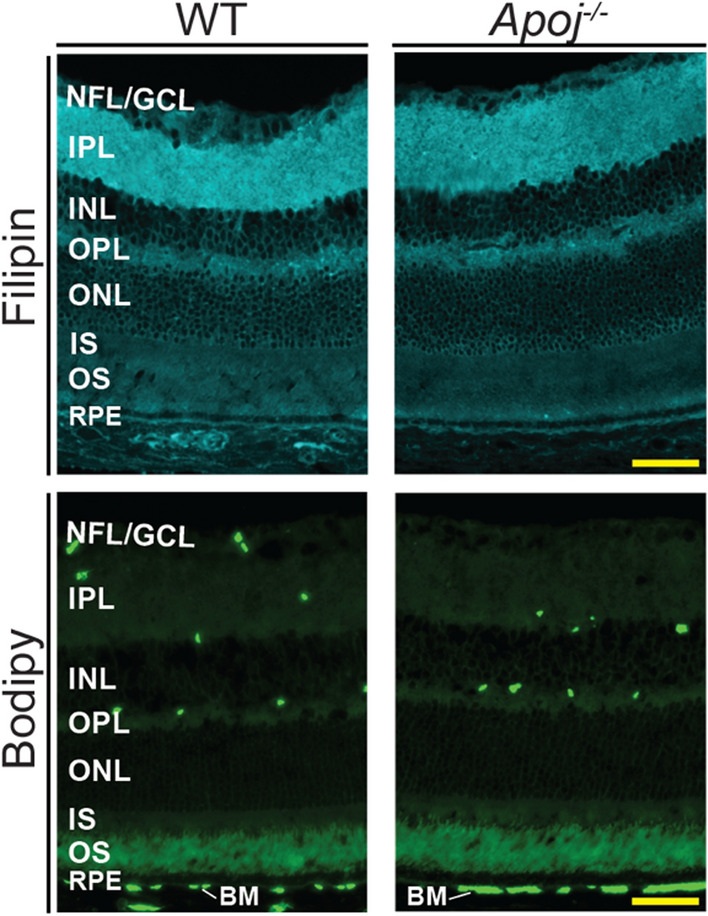
Retinal lipid distribution in one year old wild type (WT) and *Apoj*^*−/−*^ mice. Retinal cross sections were stained with filipin for unesterified cholesterol and Bodipy 493/503 for unesterified cholesterol, esterified cholesterol, triacylglycerides, and free fatty acids. Images are representative of 5 male mice per genotype. Scale bars are 50 μm. The retinal layer labeling is as described in Fig. 1

To examine whether a decrease in the retinal cholesterol content was a result of a focal cholesterol decrease in the *Apoj*^*−/−*^ retina, histochemistry localizations were carried out on 12 month-old animals. Staining with filipin to visualize unesterified cholesterol [63] did not reveal any particular retinal layer or location that were affected by this decrease (Fig. 3). Similarly, labeling with Bodipy to detect both unesterified and esterified cholesterol as well as other lipid species [64] was essentially undistinguishable between the WT and *Apoj*^*−/−*^ retinas; only, perhaps, Bruch’s membrane seemed to have more labeling in the *Apoj*^*−/−*^* vs* WT retinas (Fig. 3). Thus, both lipid stains suggested that the retinal cholesterol decrease in *Apoj*^*−/−*^ mice did not appear to be a result of a focal change. Alternatively, both filipin and Bodipy were not sensitive enough to detect this change in retinal cholesterol distribution.

### Intraocular pressure

**Fig. 4:**
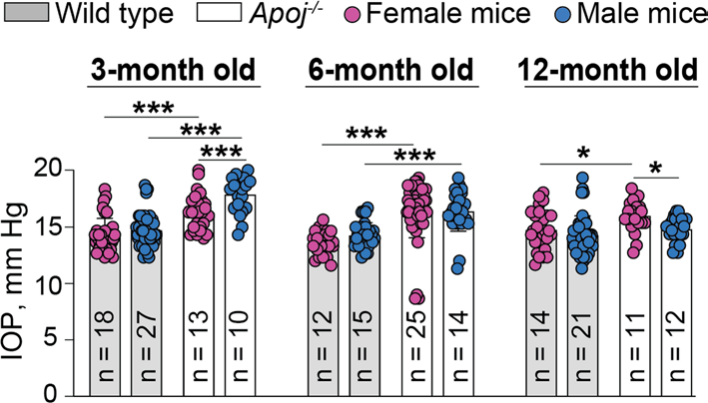
Intraocular pressure (IOP) in wild type and *Apoj*^*−/−*^ mice of different ages. Data represent the mean ± SD of the measurements in individual eyes; both eyes in each animal were evaluated. The number of animals (n) per group is indicated in the figure. *P ≤ 0.05; **P ≤ 0.01; ***P ≤ 0.001 as assessed by two-way ANOVA with Tukey's multiple comparison test

*Apoj*^*−/−*^ mice were reported to have a gradual increase in IOP from day 50 of age (the first measured time point) to day 105 of age (~ 3.5 months of age, the last measured time point) [22]. Hence, we measured the IOP in our cohort of *Apoj*^*−/−*^ mice with evaluations being at three time points: 3, 6, and 12 months of age. As compared to WT animals, there was a modest (up to 3 mm Hg) but statistically significant increase in IOP in all age groups of *Apoj*^*−/−*^ mice (either in one or both sexes) (Fig. 4). Yet, this increase did not seem to become lager with mouse age and was 2 and 3 mm Hg in 3 month old female and male *Apoj*^*−/−*^ mice, respectively; 2 and 2 mm Hg in 6 month old female and male *Apoj*^*−/−*^ mice, respectively; and 1 mm Hg and non-significant in 12 month old female and male *Apoj*^*−/−*^ mice, respectively. Thus, we confirmed a previous study [22] and began to evaluate whether there were any retinal consequences of this increased IOP.

### Retinal in vivo imaging

Elevated IOP is a risk factor for glaucoma, a group of diseases characterized by a gradual death of retinal ganglion cells (RGC) and degeneration of the optic nerve [74–76]. Structurally, these changes are manifested in the characteristic appearance of the optic nerve on fundus photos (a so-called disc cupping) and in thinning of the retinal NFL and GCL, which contain RGC. We conducted different in vivo characterizations of the *Apoj*^*−/−*^ retina in 3-, 6- and 12 month-old mice and started with the fundus examination.

**Fig. 5:**
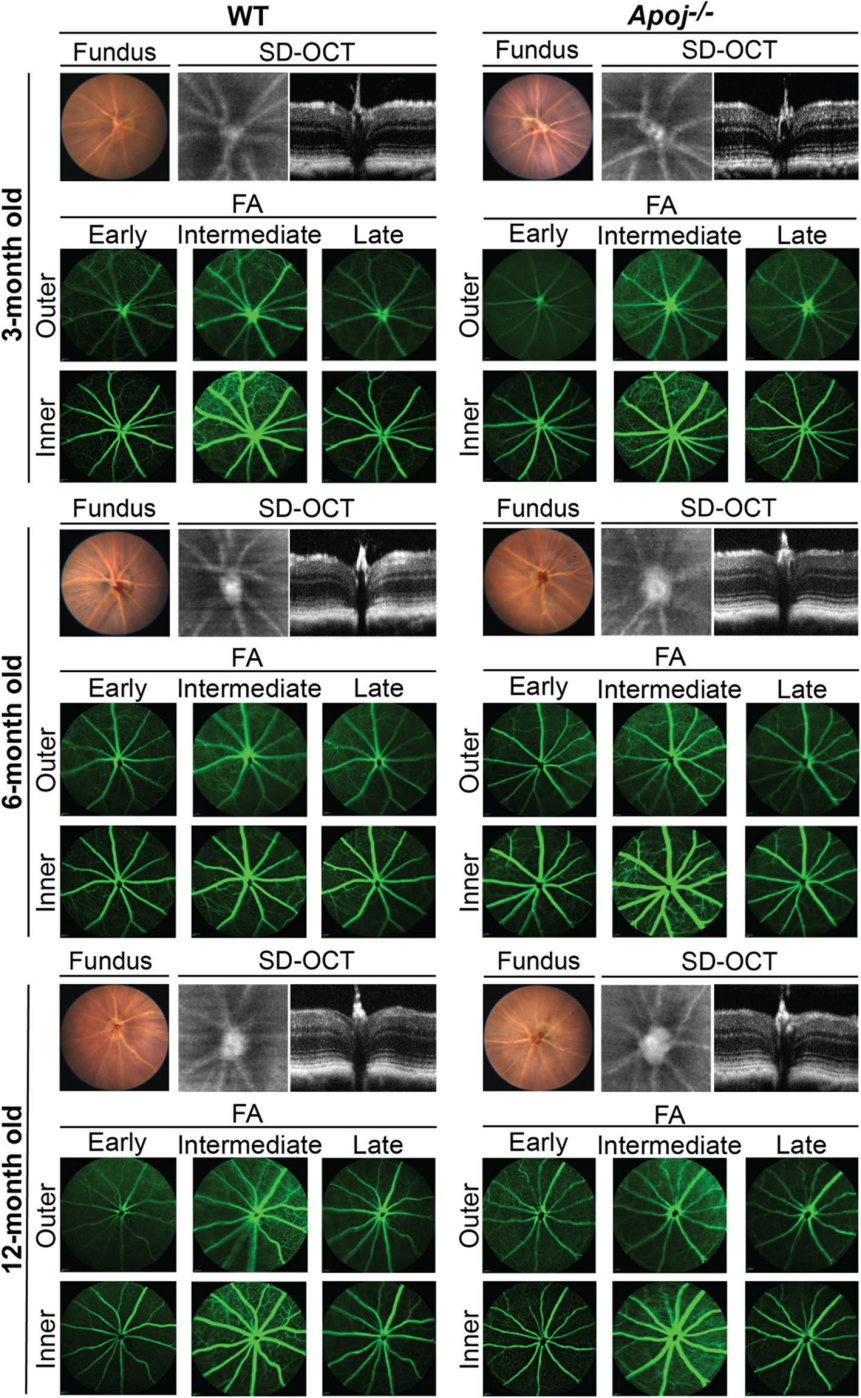
Retinal in vivo imaging in wild type (WT) and *Apoj*^*−/−*^ mice of different ages. Representative assessments (3 month old group: 5 female and 7 male WT mice and 7 female and 6 male *Apoj*^*−/−*^ mice; 6 month old group: 5 female and 5 male WT mice and 7 female and 5 male *Apoj*^*−/−*^ mice; and 12-month old group: 6 female and 5 male WT mice and 8 female and 3 male *Apoj*^*−/−*^ mice) of the same animal by fundus photography (fundus), spectral domain-optical coherence tomography (SD-OCT) and fluorescein angiography (FA). The SD-OCT panels show (from left to right) fundus imaging at the level of the retinal ganglion cell layer and retinal cross section. The FA panels show (from left to right) an early, intermediate, and late-stage fundus fluorescence. The laser beam was focused either on the outer retina or inner retina, which are nourished by the retinal and choroidal vascular networks, respectively. No sex-based differences were detected by any of the imaging modality

**Fig. 6:**
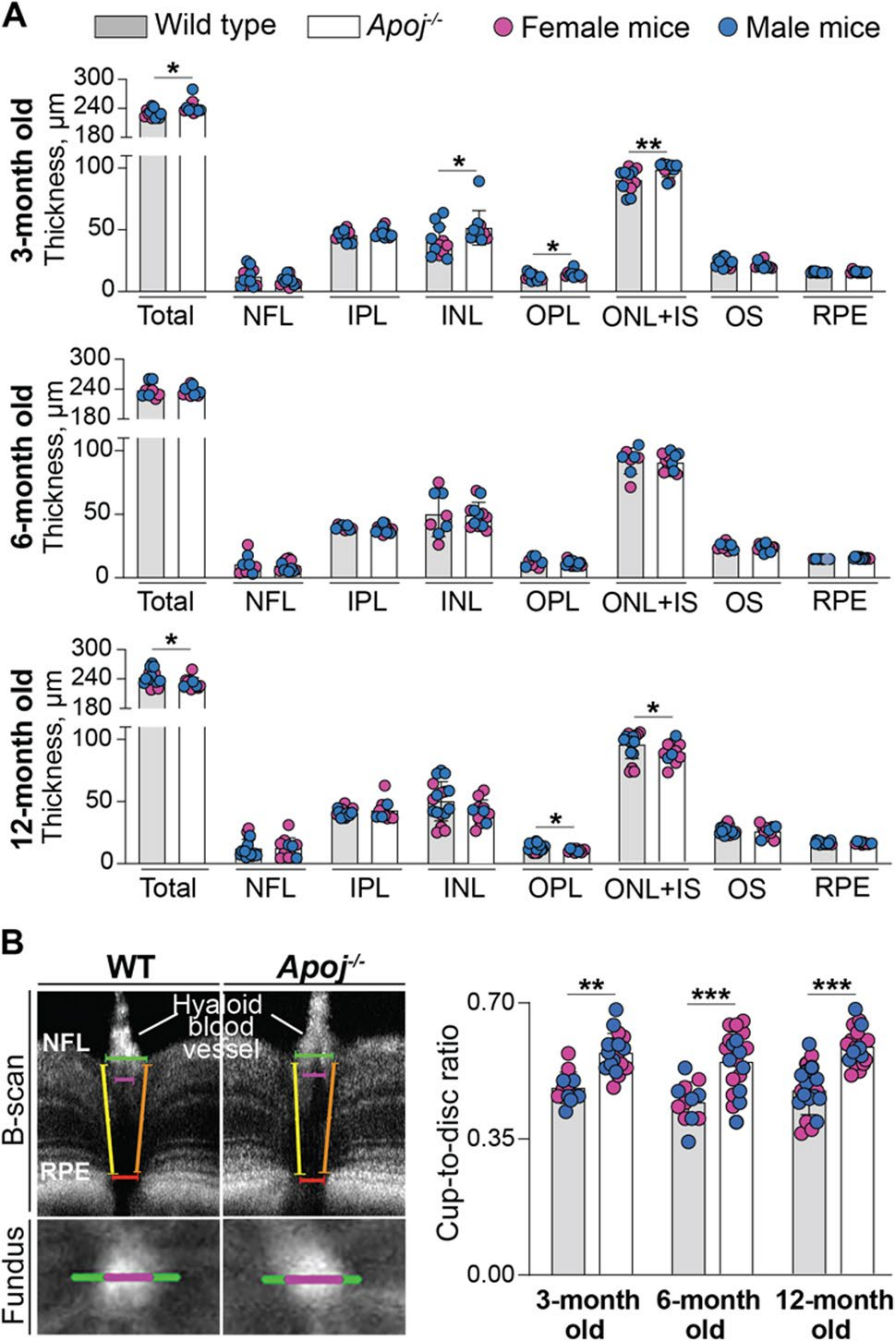
Quantifications by SD-OCT. **A** Retinal thickness in wild type and *Apoj*^*−/−*^ mice of different ages. Data represent the mean ± SD of the measurements in individual animals after the results from both eyes were averaged (3 month old group: 5 female and 7 male wild type mice and 4 female and 6 male *Apoj*^*−/−*^ mice; 6 month old group: 4 female and 4 male wild type mice and 7 female and 5 male *Apoj*^*−/−*^ mice; and 12 month old group: 8 female and 9 male wild type mice and 8 female and 3 male *Apoj*^*−/−*^ mice). *P ≤ 0.05; **P ≤ 0.01 as assessed by an unpaired non-parametric Mann–Whitney test. The retinal layer labeling is as described in Fig. 1. **B** Cup-to-disk ratio in wild type (WT) and *Apoj*^*−/−*^ mice of different ages. Left upper panels show how this ratio was measured. Specifically, the cup diameter (magenta calipers) was defined in the B-scan by the boundaries of the hyaloid blood vessel entry into the retina; the corresponding fundus images were used to support our manual selection. The disk diameter (green calipers) was determined in the B-scan by identifying the minimal distance between the retinal pigment epithelium (RPE) breakpoints in the optic nerve head (red calipers) and extending the calipers (yellow and magenta) toward the nerve fiber layer (NFL) where the nerve fibers exit the retina. Left lower panels show the horizontal cup (magenta) and disk (green) diameters in the fundus image. Right panel shows the cup-to-disk measurements. Data represent the mean ± SD of the measurements in both eyes from each animal (3 month old group: 3 female and 3 male WT mice and 4 female and 5 male *Apoj*^*−/−*^ mice; 6 month old group: 3 female and 3 male WT mice and 6 female and 4 male *Apoj*^*−/−*^ mice; and 12 month old group: 4 female and 6 male WT mice and 7 female and 3 male *Apoj*^*−/−*^ mice). *P ≤ 0.05; **P ≤ 0.01; ***P ≤ 0.001 as assessed by two-way ANOVA with Tukey's multiple comparison test

In *Apoj*^*−/−*^ mice of all age groups, the optic disc looked normal on fundus photos, although enlarged (Fig. 5), and we could not detect the characteristic for glaucoma loss of the neuroretinal rim and the optic disk cupping [74]. Therefore, ultra-high resolution SD-OCT was employed to further assess the optic disk by measuring the cup-to-disk ratio (CDR), to detect potential changes in retinal gross structure, and quantify the retinal layer thickness. In each WT and *Apoj*^*−/−*^ genotypes, the CDR values did not change with age and were higher in *Apoj*^*−/−*^ mice than WT mice (0.55–0.58 vs 0.44–0.48) (Fig. 6A), mostly because of an increase in the cup diameter with a contribution of a decrease in the disk diameter at 12 months of age.

In cross section, the retinal structure in *Apoj*^*−/−*^ mice looked normal at all ages (Figs. 5), although had changes in total thickness at different time points. As compared to WT mice, total retinal thickness was increased at 3 months of age (242 μm *vs* 229 μm), was unchanged at 6 months of age (238 μm *vs* 236 μm) and decreased at 12 months of age (231 μm *vs* 242 μm) (Fig. 6B). A decrease at 12 months of age was mainly because of a 3-μm decrease (by 23%) in the thickness of the outer plexiform layer (OPL, a synapse connection between photoreceptors to bipolar and horizontal cells), and a 6-μm decrease (by 7%) in the thickness of the layer formed by the photoreceptor somas (ONL) and IS.

Retinal blood flow and the status of the vasculature were assessed by SD-OCT Doppler flow and FA, respectively, as vascular component is suggested to contribute to glaucoma pathophysiology [77, 78]. FA was used at different post-injection times and with the laser beam focused either on the inner or outer retina. No changes were detected on SD-OCT Doppler flow (data not shown), and FA did not show any irregularities either in the retinal vasculature or vascular perfusion in *Apoj*^*−/−*^ mice of all studied ages (Fig. 5). Thus, in vivo retinal imaging documented that lack of APOJ led to the CDR increase, which did not become larger with age. This result suggested that either the observed IOP increase was not damaging enough for retinal RGC and optic nerve or the studied *Apoj*^*−/−*^ mice were not old enough to observe the RGC death and optic disk degeneration.

### Retinal RGC number

**Fig. 7:**
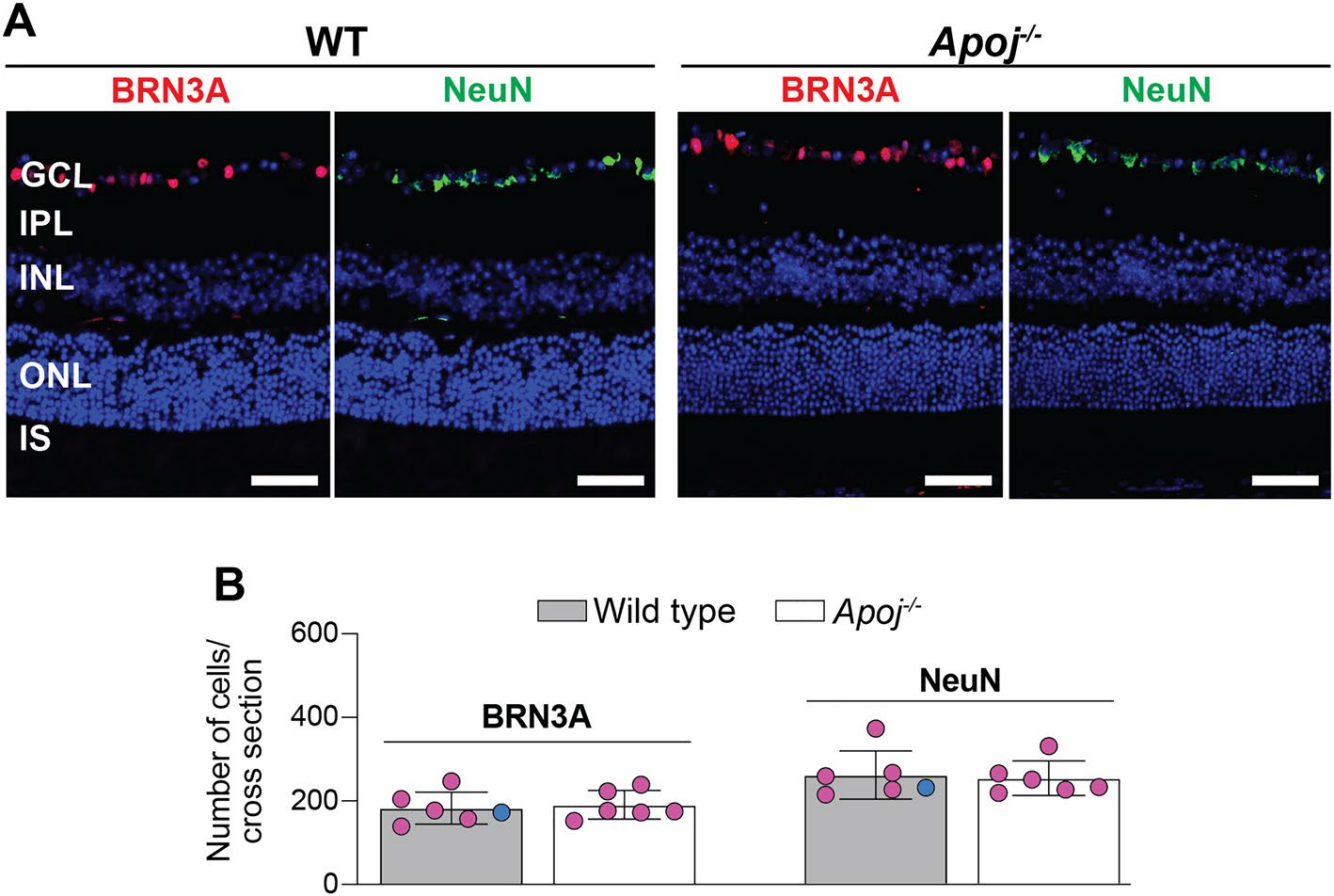
Lack of neuronal degeneration in the ganglion cell layer in 6 month old *Apoj*^*−/−*^ mice.** A** Representative images [wild type (WT) animals: 5 female and 1 male mice; *Apoj*^*−/−*^ animals: 6 female mice] of the BRN3A-positive (red) and NeuN-positive (green) cells in retinal cross sections at the levels of the optic nerve. Nuclei were stained with DAPI (blue). **B** Cell quantifications in retinal cross sections that were stained in panel A (one section per animal, n = 6) **A** Scale bars are 50 μm. The retinal layer labeling is as described in Fig. 1

This was mainly assessed in female mice by immunohistochemistry with double stains for BRN3A and NeuN as markers for the RGC and GCL, respectively, as the latter is composed of both RGC and displaced amacrine cells [79–82]. Animals were 6-month old. In *Apoj*^*−/−*^* vs* WT mice, the total count per eyecup of the BRN3A-positive cells was not changed (Fig. 7). Similarly, no difference was found between *Apoj*^*−/−*^ and WT mice in the total count per eyecup of the NeuN-positive cells. Thus, neither RGC nor GCL seemed to undergo degeneration in the *Apoj*^*−/−*^ retina at 6 months of age, consistent with in vivo imaging and retinal thickness quantifications by SD-OCT.

### Retinal RGC function

**Fig. 8:**
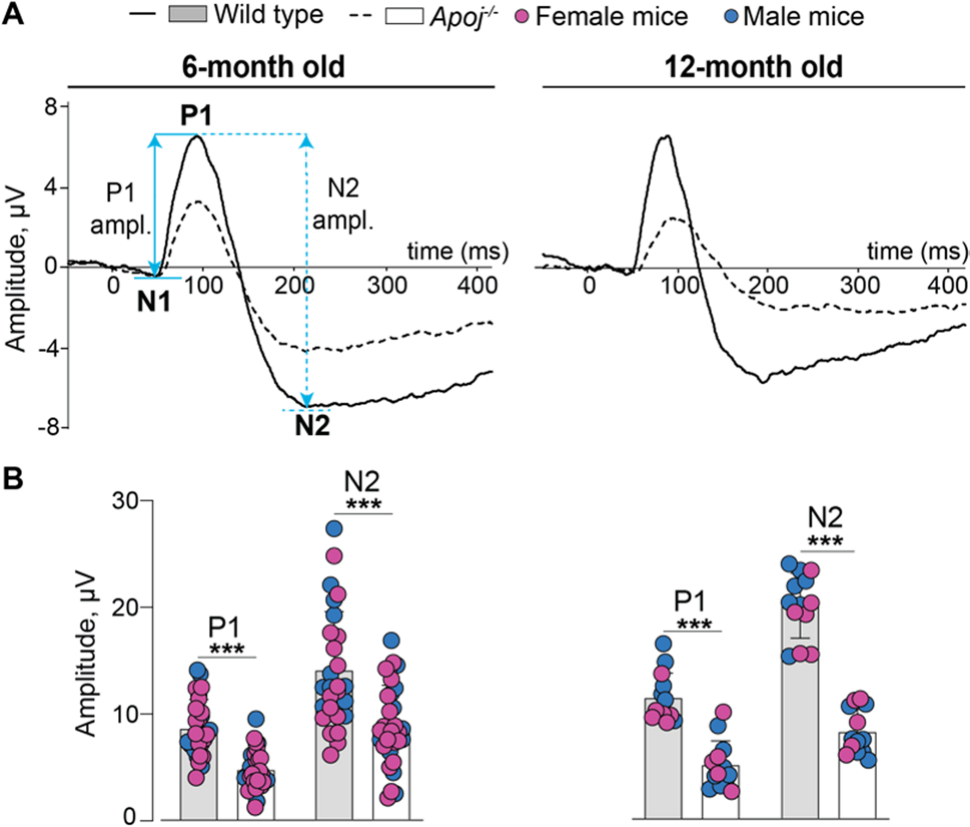
Progressive impairment of retinal function in *Apoj*^*−/−*^ mice. **A** Averaged PERG waveforms showing P1 and N2 amplitudes (ampl.) in 6- and 12 month old wild type (WT) and *Apoj*^*−/−*^ mice. **B** The quantifications of the P1 an N2 amplitudes. Data represent the mean ± SD of the measurements in one eye from each animal (6 month old group: 16 female and 11 male WT mice and 20 female and 12 male *Apoj*^*−/−*^ mice; and 12 month old group: 6 female and 7 male WT mice and 5 female and 8 male *Apoj*^*−/−*^ mice). *P ≤ 0.05; **P ≤ 0.01; ***P ≤ 0.001 as assessed by two-way ANOVA with Tukey's multiple comparison test

RGC are the output neurons of the retina as their axons extend through the optic nerve to the higher centers of the visual system in the brain. RGC dysfunction and death lead to vision impairment and ultimately blindness [83]. The RGC and optic nerve function can be evaluated by PERG, a non-invasive in vivo assessment tool, used widely in clinic and animal research, to monitor functional effects of glaucomatous neuropathy [84–86]. We recorded PERG in 6- and 12 month old mice and measured the mean amplitudes of the P1 and N2 waveform components, which both reflect the functional output of the RGC according to the manufacturer’s manual. Both P1 and N2 amplitudes were decreased in 6-month old mice *Apoj*^*−/−*^* vs* WT mice (Fig. 8B): from 8.6 to 4.7 μV or by 45% for the former, and from 14 to 8.8 μV or by 37% for the latter. The P1 an N2 amplitudes were further decreased in 12-month old mice: from 7.6 to 3.4 μV or by 55% for P1 and from 13.0 to 5.4 μV or by 59% for N2 (Fig. 8B). Thus, there was a progressive decrease with age in both PERG amplitudes in mice lacking APOJ, thus suggesting the progressive RGC/optic nerve dysfunction. This progressive dysfunction could be due to different reasons: precede the RGC death as suggested by animal models of ocular hypertension [86]; relate to the APOJ absence, which was recently identified as an astrocyte-secreted factor facilitating synapse formation and excitatory (glutamergic) synaptic transmission in the brain [87]; or reflect other, yet to identified, changes in the *Apoj*^*−/−*^ retina. Therefore, we conducted additional retinal characterizations.

### Status of retinal Muller cells and microglia

**Fig. 9:**
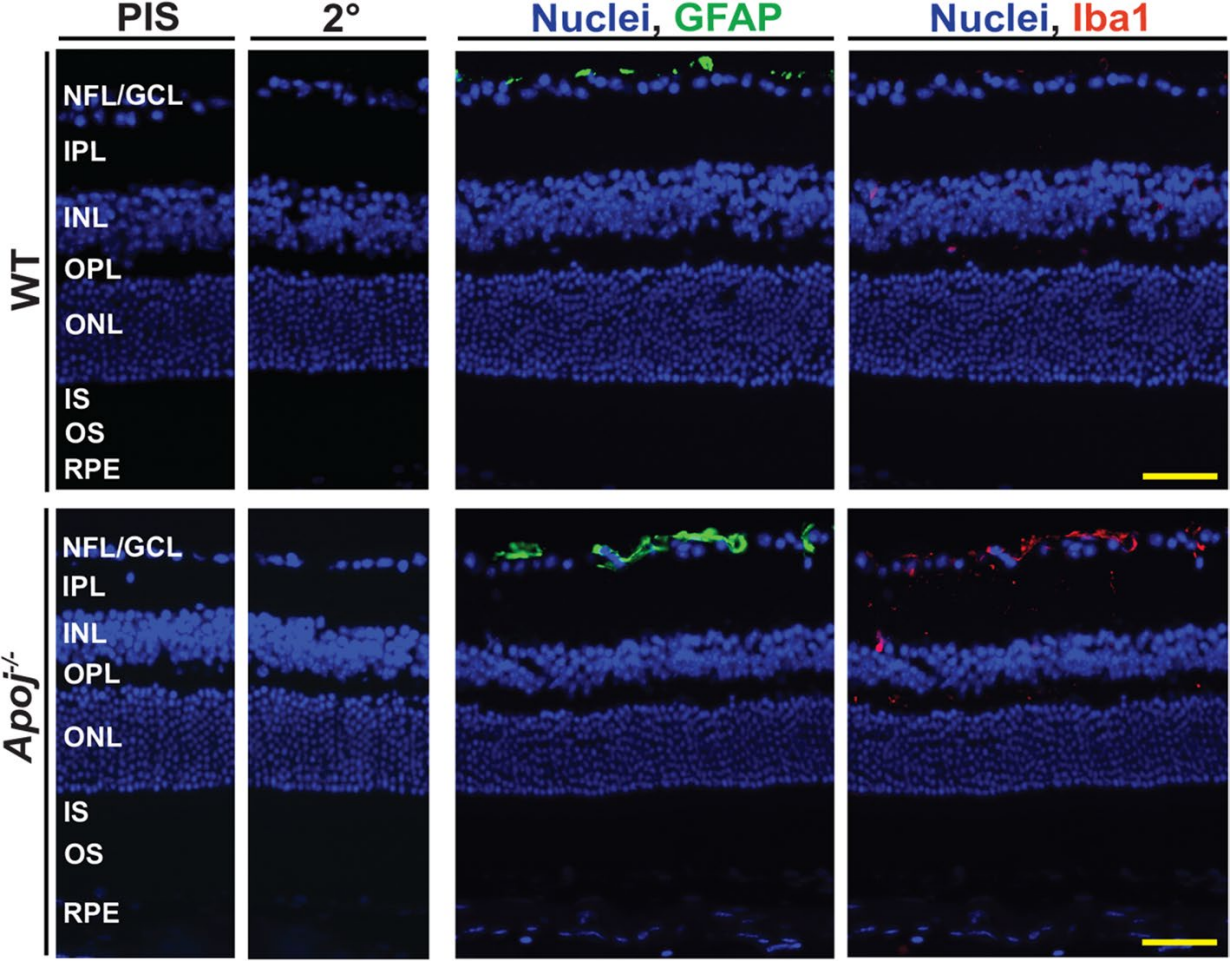
The retinal Muller cells and microglia/macrophage status in 12 month old wild type (WT) and *Apoj*^*−/−*^ mice. Representative stains (3 female and 2 male per genotype) for GFAP (green) and Iba1 (red) as markers for activated Muller cells and microglia/macrophage, respectively. Nuclei were stained with DAPI (blue). PIS, incubations with preimmune serum only; 2^0^, incubations with secondary antibody only. The retinal layer labeling is as described in Fig. 1. Yellow scale bars are 50 μm

Laser-induced IOP elevation in rats (to 34 mm Hg) was shown to activate retinal Muller and microglial cells [88]. Also, in different cell types, lack of APOJ was shown to elicit inflammatory and immune responses via the NFkB activation [89, 90]. Since both Muller cells and microglia/macrophages are important for the normal function of the RGC [91], we assessed whether these cell types were activated in the *Apoj*^*−/−*^ retina. GFAP and Iba1 were used as markers for activated Muller cells and microglia/macrophages, respectively [92, 93]. In both WT and *Apoj*^*−/−*^ retina, immunoreactivity for GFAP was only present in the NFL (the end feet of Muller cells) (Fig. 9), consistent with the predominant GFAP expression in normal retina (57). The extent of the end feet labeling seemed to be higher in the *Apoj*^*−/−*^ retina than the WT retina, possibly an apparent difference as the GFAP quantifications were not carried out due to the irregular end feet shape. Conversely, immunoreactivity for Iba1 was essentially absent in the WT retina but was more detectable in the *Apoj*^*−/−*^ retina, where the Iba1-positive cells were found in all layers, which are normally patrolled by microglia/macrophages. Specifically, most of these Iba1-positive cells were detected in the GCL and only few in the IPL, INL, and OPL. The shape of the cells was typical of resting microglia/macrophages, suggesting a lack of activation. Thus, ablation of *Apoj* and elevated IOP did not seem to significantly activate both Muller cells and microglia/macrophages and thereby contribute to the impairment of the RGC function.

### Retinal proteomics

**Fig. 10:**
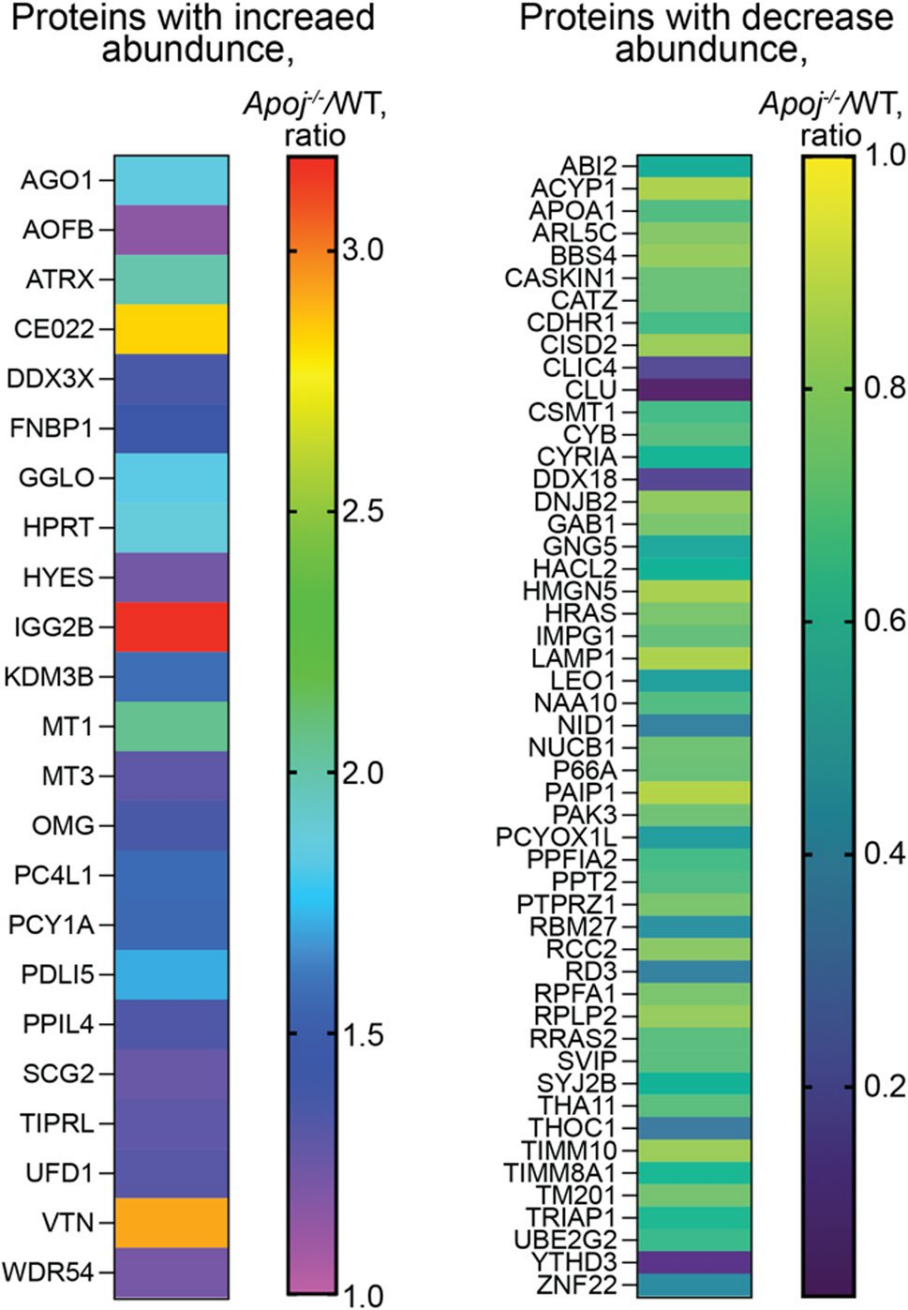
Differentially expressed proteins in the retina of 6 month old *Apoj*^*−/−*^* vs* wild type (WT) male mice. Four biological replicates per genotype, each representing a pooled sample of three retinas from three different mice were used

To gain unbiased mechanistic insights into the processes in the retina that could be affected by lack of APOJ, retinal protein abundance in *Apoj*^*−/−*^ vs WT mice was assessed by the label free approach. A total of 74 differentially expressed proteins (DEPs) were identified: 23 with increased expression and 51 with decreased expression (Fig. 10). These DEPs were then analyzed by the PANTHER software [94] for statistical overrepresentation in the gene ontology (GO) biological processes (≥ twofold enrichment, an arbitrary cut off). Of the DEP-enriched biological processes that were identified (Table 1), several were consistent with the APOJ functions already shown or suggested to be operative in the retina: retinal development (oligodendrocyte differentiation, regulation of post-synapse organization, glial cell differentiation, central nervous system development, and neurogenesis), lipid transport (lipid homeostasis), anti-apoptosis (regulation of neuron death and regulation of cell death), prevention of inflammation (regulation of NIK/NF-κB signaling), and anti-oxidative stress (regulation of response to stress) [6, 20, 21, 67, 95]. In addition, several of the identified DEP-enriched processes have not yet been confirmed to relate to the APOJ functions [4, 22, 96] in the retina: chaperone activity (ERAD pathway), vesicle-mediated transport (regulation of vesicle-mediated transport and endocytosis), actin cytoskeleton organization (regulation of actin cytoskeleton organization), cell-cycle regulation (regulation of cell cycle**)**, cell adhesion (cell–matrix adhesion and cell migration), and immune response (negative regulation of immune response). Lastly, two processes, protein phosphorylation (regulation of MAPK cascade and regulation of protein phosphorylation) as well as gene expression (negative regulation of gene expression), have not seemed to be yet suggested as the APOJ functions. Thus, the data obtained were meaningful and indicated that APOJ could play multiple roles in the retina. If so, impaired retinal function in the *Apoj*^*−/−*^ mice may not be solely due to increased IOP and disturbed retinal cholesterol homeostasis but rather reflect complex consequences of the APOJ absence on other processes in the retina.

**Table 1:** Statistical overrepresentation in the GO biological processes of the differentially expressed proteins (DEPs) in the *Apoj*^*−/−*^* vs* WT retina as identified by the PANTHER software [94]

DEPs/Total proteins	Fold enrichm	GO biological processes and DEPs involved
		**Oligodendrocyte differentiation** (GO: 0048709)
4/71	16.7	VTN, OMG, CLU, PTPRZ1
		**Regulation of postsynapse organization** (GO: 0099175)
4/110	10.8	ABI2, PDLI5, PAK3, PPFIA2
		**ERAD pathway** (GO: 0036503)
3/94	9.5	UBE2G2, UFD1, DNJB2
		**Glial cell differentiation** (GO: 0010001)
5/190	7.8	VTN, MT3, OMG, CLU, PTPRZ1
		**Cell–matrix adhesion** (GO: 0007160)
3/121	7.4	NID1, RCC2, VTN
		**Regulation of NIK/NF-κB signaling** (GO: 1901222)
2/96	6.2	AGO1, DDX3X
		**Regulation of neuron death** (GO: 1901214)
8/32	6.2	RRAS2, MT3, MT1, PAK3, HRAS, CLU, CATZ, PTPRZ1
		**Lipid homeostasis** (GO: 0055088)
3/166	5.4	DDX3X, APOA1, HYES
		**Regulation of endocytosis** (GO: 0030100)
4/237	5.0	SYJ2B, VTN, WDR54, CLU
		**Regulation of actin cytoskeleton organization** (GO: 0032956)
6/369	4.8	ABI2, APOA1, PAK3, HRAS, BBS4
		**Negative regulation of immune response** (GO: 0050777)
3/205	4.4	THOC1, UFD1, YTHD3
		**Regulation of vesicle-mediated transport** (GO: 0060627)
8/609	3.9	APOA1, LAMP1, SYJ2B, VTN, WR54, CLU, PPFIA2, IGG2B
		**Negative regulation of gene expression** (GO: 0010629)
9/954	2.8	AGO1, DDX3X, APOA1, THOC1, UFD1, YTHD3, HRAS, CATZ, BBS4
		**Central nervous system development** (GO: 0007417)
8/877	2.7	VTN, MT3, HPRT, OMG, CLU, ATRX, PTPRZ1, BBS4
		**Regulation of MAPK cascade** (GO: 0043408)
6/722	2.5	SYJ2B, MT3, WDR54, PAK3, HRAS, GAB1
		**Regulation of response to stress** (GO: 0080134)
11/1293	2.5	APOA1, TRIAP1, THOC1, LAMP1, UFD1, YTHD3, HRAS, OMG, CLU, SVIP, IGG2B
		**Regulation of cell cycle** (GO: 0051726)
8/992	2.4	DDX3X, RCC2, THOC1, TIPRL, NAA10, HRAS, ATRX, BBS4
		**Regulation of protein phosphorylation** (GO: 0001932)
9/1128	2.4	DDX3X, APOA1, VTN, MT3, HRAS, CLU, PPIL4, PTPRZ1, GAB1
		**Cell migration** (GO: 0016477)
7/899	2.3	ABI2, APOA1, VTN, TM201, SCG2, BBS4, GAB1
		**Regulation of cell death** (GO: 0010941)
13/1710	2.3	DDX3X, XTRIAP1, THOC1, RRAS2, MT3, MT1, SCG2, PAK3, HMGN5, HRAS, CLU, CATZ, PTPRZ1
		**Neurogenesis** (GO: 0022008)
10/1514	2.0	ABI2, VTN, MT3, CDHR1, HPRT, PAK3, OMG, CLU, PTPRZ1, BBS4

### EFV treatment of *Apoj*^*−/−*^ mice

Retinal 24HC, enzymatic product of CYP46A1, was suggested to be an endogenous neuroprotectant under glaucomatous conditions as this oxysterol ameliorated axonal injury and apoptotic RGC death in the ex vivo rat retina subjected to elevated (75 mM Hg) hydrostatic pressure [97, 98]. In addition, 24HC was found to be a positive allosteric activator of N-methyl-d-aspartate receptors (NMDARs) in mouse hippocampal slices [99]. The retina also expresses NMDARs, which mediate excitatory neurotransmission and play a major role in the light-evoked response of the RGC [100]. Since the 24HC levels were decreased in *Apoj*^*−/−*^ mice (Fig. 2), we treated this genotype with a small dose of EFV, a CYP46A1 allosteric activator in both brain and retina [48–50], and assessed the treatment effect on retinal sterol levels, gene expression, IOP, CDR, and PERG.

**Fig. 11:**
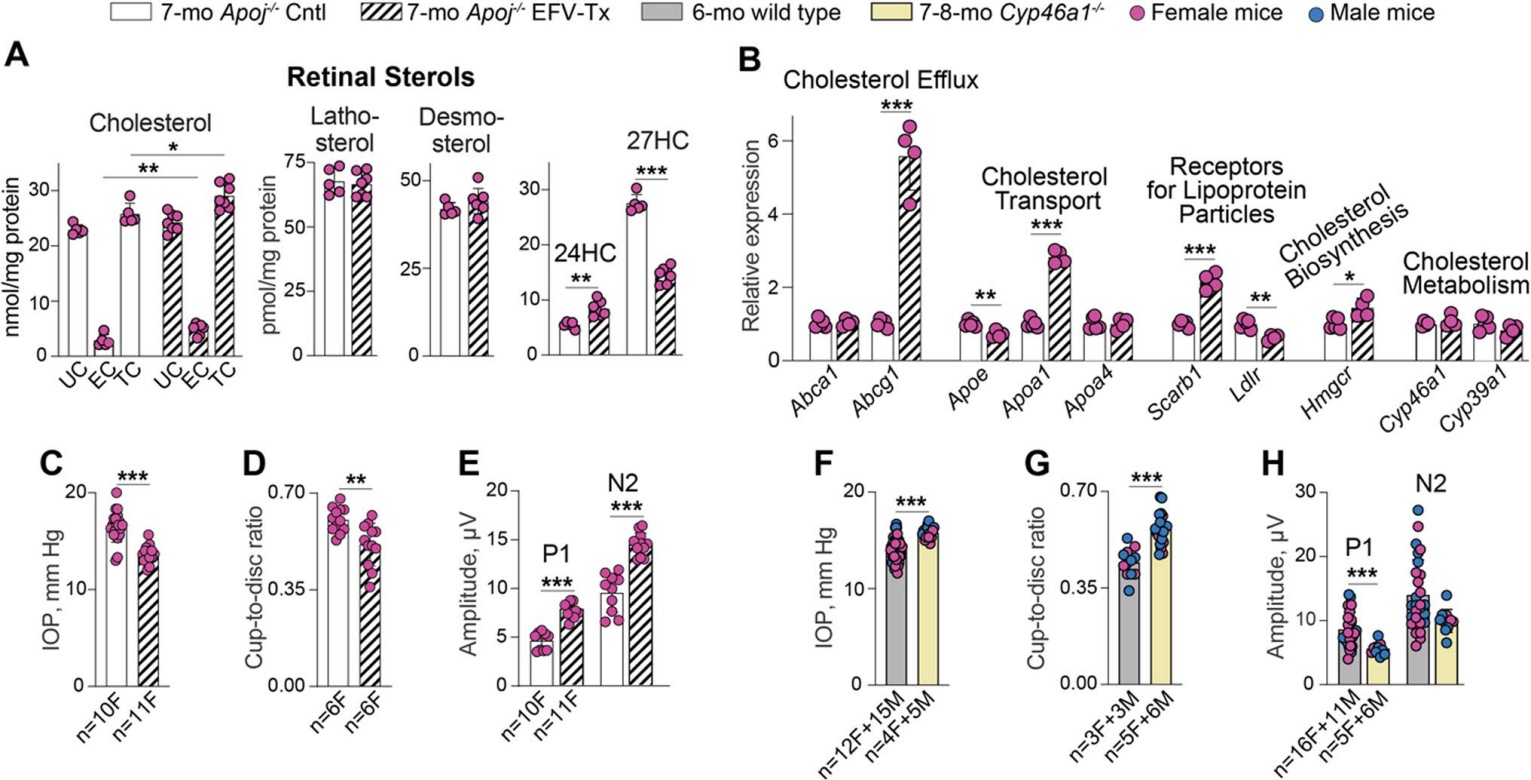
Ocular effects of EFV treatment. **A** Retinal sterol content in female *Apoj*^*−/−*^ control (Cntl) and efavirenz (EFV)-treated (Tx) mice. Data represent the mean ± SD of the measurements either in individual retinas (cholesterol, lathosterol, and desmosterol: the number of animals (n) = 5 Cntl and 7 EFV-Tx mice per each sterol group or in pooled samples (5 Cntl and 7 EFV-Tx, each containing 2 retinas from 2 different animals) for 24-hydroxycholesterol (24HC) and 27-hydroxycholesterol (27HC). **B**, retinal gene expression in female *Apoj*^*−/−*^ Cntl and EFV-Tx mice. Data represent the mean ± SD of the measurements in individual mice: n = 5 Cntl and 4 EFV-Tx. **C** and **F** intraocular pressure (IOP) in *Apoj*^*−/−*^ Cntl *vs* EFV-Tx mice and wild type *vs Cyp46a1*^*−/−*^ mice, respectively. Data represent the mean ± SD of the measurements in individual eyes; both eyes in each animal were evaluated. The number of animals and their sex (F, female; and M, male) per group are indicated in each panel. **D** and **G**, Cup-to-disk ratio in *Apoj*^*−/−*^ Cntl *vs* EFV-Tx mice and wild type *vs Cyp46a1*^*−/−*^ mice, respectively. Data represent the mean ± SD of the measurements in individual eyes; both eyes in each animal were evaluated. The number of animals and their sex per group are indicated in each panel. **E** and **H**, P1 an N2 amplitudes in the PERG waveforms in *Apoj*^*−/−*^ Cntl *vs* EFV-Tx mice and wild type *vs Cyp46a1*^*−/−*^ mice, respectively. Data represent the mean ± SD of the measurements in one eye from each animal. The number of animals and their sex per group are indicated in each panel. **F**–**H** Data for wild type mice are taken from Figs. 4, 6, 8 and are shown for comparison. *P ≤ 0.05; **P ≤ 0.01; ***P ≤ 0.001 as assessed by a two-tailed unpaired Student’s t test. No statistical significance was found between female (magenta circles) and male mice (blue circles) in the **F**–**H**; hence the data for both sexes were combined. *EC* esterified cholesterol; *IOP* intraocular pressure; *TC* total cholesterol; and *UC* unesterified cholesterol

As in our previous treatment of 5XFAD mice [50], EFV activated CYP46A1 in the *Apoj*^*−/−*^ retina as suggested by increased retinal 24HC levels, which became similar to those in 6-month old WT mice (8.2 *vs* 8.4 pmol/mg protein, Figs. 2A, 11A, please note that the sterol levels do not change with age in the two genotypes in Fig. 2A, except 27HC at 12 months of age in *Apoj*^*−/−*^ mice). Simultaneously, EFV treatment lowered retinal 27HC content to the WT levels (14.6 vs 14.4 pmol/mg protein in 6-month old WT mice, Fig. 2A, 11A), and did not affect retinal lathosterol and desmosterol levels (Fig. 11A). As a result, retinal levels of TC were increased in EFV-treated *vs* control *Apoj*^*−/−*^ mice (28.9 *vs* 25.8 nmol/mg protein) due to an increase in the EC content (4.7 *vs* 2.8 nmol/mg protein), although the UC content did not change (24.1 *vs* 22.9 nmol/mg protein) (Fig. 11A). Collectively, our sterol quantifications suggested that CYP46A1 activation by EFV likely promoted cholesterol supply to retinal neurons by the non-APOJ-containing LPP. This decreased the Muller cell 27HC levels and increased neuronal cholesterol content, subsequent 24HC production, and cholesterol esterification. Also possible is that CYP46A1 activation by EFV allowed the enzyme to compete more efficiently with CYP27A1 for cholesterol in the cells where both enzymes were expressed, thus contributing to a decrease in the retinal 27HC levels as compared to control *Apoj*^*−/−*^ mice. A decrease in the retinal 27HC levels was likely a beneficial EFV effect as 27HC was shown to be toxic to immature oligodendrocytes in the brain, favor the oligodendrocyte progenitor cell maturation, and modify protein levels in myelin [101]

To test our data interpretation, we quantified in EFV-treated *vs* control *Apoj*^*−/−*^ mice the expression of the genes which encode transporters for cellular cholesterol efflux (*Abca1* and *Abcg1*), most abundant retinal apolipoproteins (*Apoe*, *Apoa1*, and *ApoA4*), cell surface receptors for bi-directional (in and out) cholesterol transport (*Scarb1*) and uptake on LPP (*Ldlr*) as well as enzymes involved in cholesterol biosynthesis (*Hmgcr*) and metabolism (*Cyp46a1* and *Cyp39a1*). Indeed, EFV treatment significantly increased the expression of *Abcg1, ApoA1*, and *Scarb1*, which pertain to LPP formation and receptor-mediated LPP uptake, as well as the expression of *Hmgcr*, which plays a key role in cellular cholesterol biosynthesis (Fig. 11B). The expression of another LPP apolipoprotein *Apoe* was modestly decreased as was the expression of the receptor for LDL (*Ldlr*). These data were consistent with EFV treatment upregulating retinal formation and uptake of APOA1-containing LPP. Furthermore, the expression of *Cyp46a1* and *Cyp39a1*, which converts 24HC to 7α,24-dihydroxycholesterol, was unaffected, consistent with increased 24HC formation in EFV-treated *vs* control *Apoj*^*−/−*^ mice (Fig. 11A) being indeed due to allosteric activation of CYP46A1 by efavirenz.

With respect to glaucoma risk factors, EFV treatment decreased, as compared to the control *Apoj*^*−/−*^ group, the IOP from 17 to 14 mm Hg and CDR from 0.60 to 0.51, and increased the amplitudes of P1 (from 4.6 to 7.7 μM) and N2 (from 9.5 to 14.0 μM) in the PERG waveforms (Fig. 11C,D,E). As compared to 6-month old WT mice (Figs. 4,6,8), EFV treatment of *Apoj*^*−/−*^ mice normalized their IOP (14 *vs* 14 mM Hg), CDR (0.51 *vs* 0.44), and the N2 amplitude (14 *vs* 14 μM) and improved the P1 amplitude (7.7 *vs* 8.6 μM). The PERG changes supported the 24HC role as a positive allosteric activator of NMDARs in the RGC.

To obtain support that the observed improvements in the glaucoma risk factors in EFV-treated *Apoj*^*−/−*^ mice were mediated by CYP46A1 activation, we evaluated *Cyp46a1*^*−/−*^ mice. As compared to WT animals, *Cyp46a1*^*−/−*^ mice had increased IOP and CDR and decreased amplitudes of the PERG waveforms (Fig. 11F, G, H), i.e., changes opposite to those elicited by CYP46A1 activation and consistent with the beneficial CYP46A1 role under the glaucomatous conditions [97, 98].

## Discussion

Studies of *Apoj*^*−/−*^ mice provided several novel findings. First, they documented the APOJ importance in retinal cholesterol homeostasis. Second, they supported the APOJ-glaucoma link. Third, they revealed the APOJ-24HC connection and highlighted the role of 24HC and its producing enzyme CYP46A1 for RGC function.

A decrease in the total retinal cholesterol content in *Apoj*^*−/−*^ mice is a very unusual finding as in all our previous studies of mice with ablation of cholesterol-related genes, retinal cholesterol content was either unchanged (*Apod*^*−/−*^ and *Soat1*^*−/−*^ mice) or increased (*Apoe*^*−/*−^, *Apod*^*−/−*^*Apoe*^*−/−*^*, Cyp27a1*^*−/−*^, *Cyp46a1*^*−/−*^, *Cyp27a1*^*−/−*^*Cyp46a1*^*−/−*^, *Cyp27a1*^*−/−*^*Cyp46a1*^*−/−*^* Soat1*^*−/−*^, and *Cyp27a1*^*−/−*^*Cyp46a1*^*−/−*^*Apoe*^*−/−*^ mice) [35, 42, 45, 60, 61, 102]. Such an unusual effect of the apolipoprotein absence could be due to the APOJ important role in delivering cholesterol from Muller cells to retinal neurons including RGC. Indeed, Muller cells constitute the main macroglia of the retina with their branching end feet wrapping around RGC somas and blood vessels; fine lateral processes ramifying throughout the synaptic layers; apical processes wrapping around the photoreceptor cell bodies; and finger-like microvilli contacting the photoreceptors [103]. Muller cells seem to highly express HMGCR enzyme [31], the rate-limiting enzyme in the cholesterol biosynthesis, and also express a number of genes related to cellular cholesterol export (e.g., *Abca1*, *Abcg1*, *Apoe*, and *Scarb1)* and cholesterol homeostasis regulation (e.g., *Lxr* and *Srebp2*) [104, 105]. Accordingly, Muller cells were suggested to be the major cholesterol providers for the retina [20, 31, 41, 104, 106]. Also, in cell culture, Muller cells were shown to synthesize and secrete cholesterol-rich APOJ and APOE-containing LPP, and similar-sized LPP were found in mouse vitreous in contact with the axons of RGC, which run along the surface of the vitreous [20]. Collectively, these data suggested that the APOJ- and APOE-containing LPP deliver cholesterol from Muller cells to RGC [20].

Our APOJ immunolocalizations in Muller cells and RGC (Fig. 1) and retinal sterol quantifications in *Apoj*^*−/−*^ mice (Fig. 2A) are consistent with this proposed APOJ role and indicate that cholesterol transport on APOJ-containing LPP from Muller cells to neurons (RGC) cannot be compensated by transport on retinal LPP formed by other apolipoproteins, for example by APOE, which Muller cells also produce and assemble into LPP [20]. This interpretation is supported by genetic *Apoe* ablation, which had an opposite effect, as compared to the *Apoj* ablation, on animal retinal sterol profile. The levels of cholesterol, lathosterol, and desmosterol became increased by at least twofold and the 24HC levels remained unchanged in *Apoe*^*−/−*^ mice [42]. Thus, LPP formed by APOJ and APOE seems to transport cholesterol to different cell types and their roles in the retina are not redundant. Perhaps LPP containing APOA1 could deliver cholesterol to RGC in APOJ absence. This would be consistent with ubiquitous retinal APOA1 expression [43, 107], lower APOA1 expression and TC levels in the *Apoj*^*−/−*^* vs* WT retina (Figs. 2A,10), and increased *Apoa1* expression and TC levels in EFV-treated *vs* control *Apoj*^*−/−*^ mice (Fig. 11B). Further studies are necessary to ascertain retinal APOA1 roles.

In addition to the striking effect on retinal cholesterol maintenance, the present study supported the APOJ-glaucoma link. Previously, APOJ was investigated as an extracellular chaperone, preventing the precipitation and aggregation of misfolded extracellular proteins [108], and several *APOJ* SNPs were assessed as risk factors for exfoliation syndrome (XFS) and exfoliation glaucoma (XFG), a secondary glaucoma type. The rationale for the latter studies was that APOJ is a component of exfoliation material, and its expression is altered in XFS and XFG [19]. Currently, the *APOJ*-XFS/XFG association results are not consistent but they do not exclude the possibility of *APOJ* involvement in complex gene–gene and gene-environment interactions, thus contributing to the pathogenies of XFG [19]. Also, in agreement with the chaperone role, a recent study of *Apoj*^*−/−*^ mice showed that APOJ is a modulator of IOP via the regulation of actin cytoskeleton and extracellular matrix in the trabecular meshwork [22]. Herein, we confirmed an IOP increase in *Apoj*^*−/−*^ mice and showed that this genotype has two additional glaucoma risk factors – CDR and impaired RGC function. (Figs. 4, 6B, 8) The latter was progressive and was not due to the RGC degeneration, at least at 6 months of age (Fig. 7).

So far, two mechanisms were suggested for the neuronal APOJ effects. In the first, APOJ was identified as an astrocyte-derived synaptogenic (increased excitatory synapse formation) and anti-amyloid factor that promoted excitatory but not inhibitory synaptic transmission in the brain [87]. In the second mechanism, APOJ was shown to be as a stress-response secretory chaperone and a modulator of IOP via the actin-based contractile machinery as well extracellular matrix production, secretion, and fibrogenic function in the trabecular meshwork [22]. In the present work, we demonstrated that the RGC function was impaired in *Apoj*^*−/−*^ mice and that their retinal levels of 24HC, the positive allosteric activator of NMDARs on RGC [99, 100], were reduced as compared to WT animals (Fig. 2A). Notably, we showed that these changes were rescued by treating *Apoj*^*−/−*^ mice with EFV, which activated CYP46A1 and increased retinal 24HC production as well as improved RGC function (Fig. 11A, E). Moreover, we showed that the RGC function was impaired in *Cyp46a1*^*−/−*^ mice (Fig. 11H) and thus, obtained in vivo results that collectively provided strong evidence for the third potential mechanism of the neuronal APOJ effects. This mechanism could be via the APOJ-mediated cholesterol supply to retinal neurons for subsequent 24HC production by CYP46A1 to allosterically activate NMADR. This proposed third mechanism may be of relevance to individuals carrying the frequent rs2279590 *APOJ* polymorphism (minor allele frequency in Caucasians is ~ 40%), a risk factor for both Alzheimer’s disease and XFG; frequent rs11136000 and rs9331888 (minor allele frequencies in Caucasians are 30–40%), risk factors for only Alzheimer’s disease; as well as less frequent rs3087554 (minor allele frequency in subjects of Northwestern European descent is 7%) associated only with XFG [15, 19, 109, 110].

Can the overall reduction in retinal UC and TC be the fourth mechanism, by which APOJ exerts its effect on neuronal function? Cholesterol is known to be abundant is plasma membranes and synaptic vesicles and was shown to modulate multiple aspects of neurotransmission. These include the biogenesis of synaptic vesicles, protein organization in synaptic vesicles, and synaptic vesicle exocytosis at the presynapse [111–113]; abundance of SNAREs, a class of integral membrane proteins involved in synaptic vesicle fusion [114, 115]; NMDA-induced long-term depression, and memory formation in mice with memory deficits [116]; excitatory postsynaptic currents and long-term potentiation [117–119]; endocytosis and internalization of AMPA receptors, consequently impairing learning and memory [116], and finally synapse development [120, 121]. Accordingly, yes, a reduction in retinal UC and TC can in principle be the fourth mechanism of the APOJ effect on neuronal function. The caveat is that it is currently unknown to what extent the levels of neuronal cholesterol must be changed to see the effect on neurotransmission, and whether there is a threshold in the change of neuronal cholesterol levels that can be tolerated by neurons. In the case of *Apoj*^*−/−*^ mice, the maximal reductions in retinal UC and TC were 21% and 36%, respectively (Fig. 2A), but the corresponding reductions in retinal neurons were not determined due to lack of methodologies to conduct such quantifications. EFV treatment did not fully restore retinal TC levels, although increased them by 12% and improved RGC function (Fig. 11H). Future studies are required to ascertain whether APOJ affects RGC function via a reduction of retinal cholesterol.

As for an increased IOP and CDR (possibly secondary to the IOP change), the normalizing EFV treatment effect on these glaucoma risk factors in *Apoj*^*−/−*^ mice is consistent with the available literature data. Animal models of glaucoma showed that elevated IOP induces either a transient increase in retinal CYP46A1 but not 24HC levels (laser photocoagulated eyes) [122] or a pressure-dependent increase in both retinal CYP46A1 expression and 24HC levels (ex vivo retina under high pressure) [97]. In the latter model, increased CYP46A1 expression was predominantly localized to the RGC, while also present in the IPL and OPL composed of synapses. Furthermore, exogenously administered 24HC ameliorated pressure-induced structural and neuronal retinal damage, whereas voriconazole, a CYP46A1 inhibitor, was severely toxic even at a normobaric pressure [97]. Collectively, these data suggested that increased CYP46A1 expression and 24HC production in the retina under glaucomatous conditions have a protective effect [97].

Genetic studies provide further support for ocular metabolism of cholesterol as a process of relevance to glaucoma. A polymorphism (rs754203) in *CYP46A1* was proposed to be a risk factor for primary open-angle glaucoma (POAG) [123], but a subsequent study did not replicate this association [124]. Yet, two investigations found that functionally deficient mutations in *CYP39A1*, which metabolizes the CYP46A1 product 24HC, were significantly associated with XFS [125, 126]. Carriers of these mutations had a twofold increased risk of XFS with EC accumulations within the exfoliation deposits on the surface membrane of the ciliary epithelium [125]. Apparently, abnormally high 24HC levels in the *CYP39A1* SNP carriers likely inhibit CYP46A1 activity and hence cholesterol elimination, thus leading to cholesterol esterification and EC accumulation. Notably, carriers of the more severe loss-of-function CYP39A1 G204E mutation had higher rates of glaucoma and a more severe glaucoma with a higher likelihood of laser or glaucoma surgery. Also, the CYP39A1 G204E carriers had almost a tenfold increased risk for glaucoma-related blindness and a shorter time until blindness (13.7 months) as compared to patients with XFS, who did not carry any CYP39A1 mutations (105.9 months) [126].

Thus, studies by us and others support the protective CYP46A1 role under the glaucomatous conditions and suggest that pharmacologic CYP46A1 activation with low-dose EFV could be beneficial. Obviously, tests on animal models of glaucoma treated with low-dose EFV are needed to test this inference. These tests are warranted by lack of approved glaucoma treatments that directly target RGCs and now only include treatments that are indirectly protective for RGCs by lowering IOP. Moreover, animal studies suggest that administration of low dose EFV could represent a disease-modifying treatment for Alzheimer’s disease [48, 49, 127] by affecting multiple and apparently unlinked processes (e.g., lipid raft formation, protein phosphorylation, synaptic glutamate release, gene transcription, energy metabolism, autophagy, apoptosis, and other). These processes could be united by the CYP46A1 activation effects on physico-chemical properties of plasma membranes, acetyl-CoA production, and mevalonate formation during cholesterol biosynthesis [55, 56]. Testing of low-dose EFV has already been conducted in a small clinical study of patients with early Alzheimer’s disease and did not raise any safety concerns [57]. Thus, treatment with EFV could provide an additional therapeutic effect for subjects with glaucoma risk factors who have Alzheimer’s disease.

In summary, ocular evaluations of *Apoj*^*−/−*^ mice demonstrated important APOJ roles in retinal cholesterol homeostasis, normal IOP maintenance, and RGC function. These evaluations, along with EFV treatment and characterization of *Cyp46a1*^*−/−*^ mice, revealed the novel APOJ-24HC/CYP46A1 link and suggested a principally new therapeutic approach for reducing glaucoma risk factors based on CYP46A1 activation with low-dose anti-HIV drug EFV.

## Data Availability

All the original data related to the figures in this article are available without undue reservation upon reasonable request to the corresponding author.

## Abbreviations

7HCA: 7α-Hydroxy-3-oxo-4-cholestenoic acid
24HC: 24-Hydroxycholesterol
27COOH: 5-Cholestenoic acid
27HC: 27-Hydroxycholesterol
APOJ: Apolipoprotein J
CDR: Cup-to-disk ratio
EC: Esterified cholesterol
EFV: Efavirenz
FA: Fluorescein angiography
GCL: Ganglion cell layer
GFAP: Glial fibrillary acidic protein
Iba1: Ionized calcium binding adaptor molecule
INL: Inner nuclear layer
IOP: Intraocular pressure
IS: Inner segments
LPP: Lipoprotein particles
NFL: Nerve fiber layer
NMDAR: N-methyl-d-aspartate receptors
OS: Outer segments
PERG: Pattern electroretinography
PBS: Phosphate buffer saline
RGC: Retinal ganglion cells
RPE: Retinal pigment epithelium
SD-OCT: Spectral domain optical coherence tomography
UC: Unesterified cholesterol
WT: Wild type
XFG: Exfoliation glaucoma
XFS: Exfoliation syndrome
